# Adolescents display distinctive tolerance to ambiguity and to uncertainty during risky decision making

**DOI:** 10.1038/srep40962

**Published:** 2017-01-18

**Authors:** Wouter van den Bos, Ralph Hertwig

**Affiliations:** 1 Center for Adaptive Rationality, Max Planck Institute for Human Development, D-14195 Berlin, Germany

## Abstract

Although actuarial data indicate that risk-taking behavior peaks in adolescence, laboratory evidence for this developmental spike remains scarce. One possible explanation for this incongruity is that in the real world adolescents often have only vague information about the potential consequences of their behavior and the likelihoods of those consequences, whereas in the lab these are often clearly stated. How do adolescents behave under such more realistic conditions of ambiguity and uncertainty? We asked 105 participants aged from 8 to 22 years to make three types of choices: (1) choices between options whose possible outcomes and probabilities were fully described (choices under risk); (2) choices between options whose possible outcomes were described but whose probability information was incomplete (choices under ambiguity), and (3) choices between unknown options whose possible outcomes and probabilities could be explored (choices under uncertainty). Relative to children and adults, two adolescent-specific markers emerged. First, adolescents were more accepting of ambiguity; second, they were also more accepting of uncertainty (as indicated by shorter pre-decisional search). Furthermore, this tolerance of the unknown was associated with motivational, but not cognitive, factors. These findings offer novel insights into the psychology of adolescent risk taking.

Adolescence is a perplexing period of human development. It is a time of good health and great strength. Yet mortality and morbidity rates also rise and peak during the adolescent years^1^,^2^,^3^,^4^. One key reason for this darker side of adolescence is that young people are more likely than children or adults to engage in risky and impulsive behaviors such as reckless driving, binge drinking, unprotected sex, and experimenting with drugs^3^,^4^,^5^,^6^. Regulatory and one-size-fits-all policy interventions to contain potentially hazardous behaviors have had limited success, as have educational programs^7^,^8^,^9^,^10^. More effective interventions could help adolescents to better navigate the opportunities and perils of their environments. However, developing such interventions would require, among other things, a more nuanced understanding of a puzzling discrepancy in the available evidence.

A recent meta-analysis found a striking deviation between the trajectories of adolescent risk-taking behaviors observed in the laboratory and those seen in real life^11^. Specifically, experimental studies often report a linear decrease in risky behavior from childhood to adulthood^11^. However, these findings are at odds with actuarial data as well as with adolescents’ self-reported risk attitudes^12^. What could explain this incongruity? One possibility is that the behaviors elicited by many experimental tasks are an inadequate proxy for real-world risk behavior^13^. Perhaps the most frequently implemented choice situation in the lab is referred to as *choice under risk*^14^,^15^. In these experiments, which often involve monetary gambles, all possible outcomes and probabilities are fully described. When adolescents use drugs or engage in unprotected sex, in contrast, they may have only a vague idea of the possible consequences of their actions and the likelihoods of those consequences. Furthermore, they may have the proverbial opportunity to “look before they leap.” That is, they may be able to search for information, hence reducing some uncertainty, before embarking on an activity. For instance, they may actively probe their peers’ experience or simply observe what happens to others engaging in a risky activity. In other words, they may explore their options without directly experiencing (un) desired outcomes.

Most classic experiments do not capture these two key aspects: decision makers’ incomplete knowledge of likelihoods and the exploratory agency allowed by many real-world situations. However, a frequently implemented experimental choice situation that does aim to mimic incomplete knowledge offers incomplete descriptions of outcomes’ probabilities, thus eliciting *choice under ambiguity*^16^ (see Fig. 1). Camerer^17^ defined ambiguity as the “*known-to-be-missing* information” (p. 654). In these experiments, individuals are free to choose but they are not at a liberty to search for more information than is given. Another experimental choice situation aims at capturing exploratory agency. Here, individuals start out completely ignorant of outcomes and probabilities but are invited to learn about them—and ultimately make decisions based on their experience of the options’ outcomes and likelihoods^18^. These experienced-based choices represent situations in which the outcome space is only partially known, meaning that a not-yet-experienced outcome—the metaphorical ‘black swan’—may still be lurking somewhere. In this sense, decisions from experience are distinct from choices under ambiguity, in which information is “known to be missing.” Furthermore, decision makers are enabled to control the degree of uncertainty they can tolerate, whereas the classic ambiguity (or risk) task does not support such agency. In order to mark this difference, we refer to these types of decisions as *choices under uncertainty.*

What is known about the development of individual attitudes to risk and ambiguity? The majority of adults across myriad risky choice studies have emerged to be *risk averse*^19^. When given the choice between options with equal expected values, they chose the option with the smaller outcome variance. Most adults also tend to be ambiguity averse. That is, they prefer *known* risks (e.g., an urn of 50 red and 50 green balls, with a reward for drawing a green ball) to *unknown* risks (e.g., an urn with 100 red and green balls in some unknown combination)^16^,^20^. Furthermore, adults’ risk and ambiguity attitudes do not appear to be correlated^21^. At the same time, two recent studies suggest that adolescents are more ambiguity seeking than adults^20^,^22^ (but see ref. 23), and another study suggests that young children may be ambiguity neutral^24^. However, no previous studies have elicited the choice behaviors and attitudes of pre-adolescent, adolescent, and adult decision makers within the same choice situation as well as between choice situations. Consequently, it remains unclear whether ambiguity tolerance peaks in adolescence or, like risk attitude as measured in the laboratory, reveals a linear developmental trend starting in childhood.

What is known about the development of search behavior in decisions under uncertainty? One signature finding from the adult literature is that individuals tend to sample relatively little information before making a consequential decision^18^, thus increasing the likelihood that low-probability (rare) outcomes remain unknown. How do adolescents behave when permitted to explore and thus attenuate uncertainty? To date, there has been no developmental research on the amount of search in experienced-based choice. However, in light of adolescents’ propensity to act impulsively, one might expect adolescents to sample even less than adults. That is, tolerance of uncertainty—in terms of lack of knowledge about the outcome space (i.e., possible outcomes) and the likelihood of potential outcomes—may be even more pronounced in adolescents than in adults.

No previous developmental investigation has *concurrently* investigated risk, ambiguity, and uncertainty attitudes from late childhood to young adulthood. To fill this gap, we investigated a developmental population ranging from late childhood to young adulthood (8‒22 years). This approach made it possible to characterize developmental trends preceding, during, and following adolescence. Our first question was this: Do individuals’ risk, ambiguity, and uncertainty attitudes—measured in terms of the preference for safe (vs. risky) and known-risk (vs. ambiguous) options and sampling effort—obey parallel or distinct developmental trajectories? Specifically, do these attitudes follow (1) a linear/monotonic trend, (2) a quadratic trend peaking in adolescence, or (3) an asymptotic trend, indicating a changing attitude between childhood and adolescence that stabilizes in early adulthood? Our second question concerned the extent to which the trajectories of risk, ambiguity, and uncertainty attitudes are synchronous with the developmental trajectories of, first, core cognitive processes (cognitive ability, working memory, and approximate number sense), and, second, affective and motivational states (sensation seeking) that may be involved in the processing of the respective choice tasks. Third, we examined how closely the three attitudes correlated with potentially harmful real-world behavior. To this end, we collected self-report data on respondents’ risky behaviors^25^.

## Results

To be able to identify developmental trends, we tested 105 participants (53 female) between the ages of 8 and 22 years (*M* = 14.87, *SD* = 4.27). The sample was divided into age bins, each containing 7 subjects. Within the bins, the gender difference was never more than one. For the subset of participants aged between 8 and 18 years, we collected parental income^26^ as a proxy for the household’s socioeconomic status. We found no age-related differences in estimated socioeconomic status, nor was it correlated with any of the other variables of interest (see Supplementary Information).

We were interested in whether developmental trends in risk-taking behavior across this age range were linear, quadratic (peaking in early/mid-adolescence), or asymptotic (changing between childhood and adolescence but stabilizing in early adulthood). To address this question, we performed independent linear regressions incorporating one of the three age regressors and all significant age trends (see ref. 27). If there was more than one significant trend, we applied Bayesian model comparison to assess the relative quality of the statistical models given the data.

**Figure 1:**
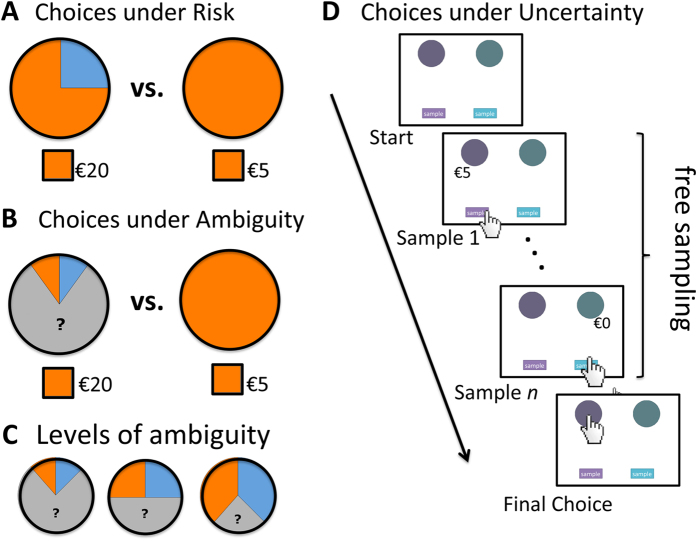
Experimental tasks. (**A**) Choices under risk were represented by a wheel of fortune consisting of 10 slices of different colors. The orange slices were associated with possible earnings or losses; if the spinner stopped at the blue slices, the player would receive nothing. (**B)** Choices under ambiguity were represented in exactly the same way as the risky gambles, but this time (gray) occluders on top of the wheels hid part of the information. (**C)** Three levels of ambiguity were implemented. (**D**) In the choices under uncertainty task, participants were able to sample from two payoff distributions before making a final choice. Participants sampled by pressing the sample button and chose by clicking on the corresponding circle. All experimental tasks were self-paced.

Each participant was presented with the three choice situations (see Fig. 1). Choices under risk and choices under ambiguity, which were both description-based, were randomly intermixed; choices under uncertainty were based on individuals’ sequential experience. The order of description- and experienced-based blocks was randomized across participants. Order had no effect on the reported results. Outcome valence is known to be an important modulator of developmental trends: Relative to adults, children and adolescents have shown high loss sensitivity in probabilistic learning and experience-based tasks^28^,^29^,^30^. More recently, imaging studies have found that adolescents have heightened gain sensitivity in description-based paradigms^31^,^32^,^33^,^34^. We therefore included gain and loss lotteries in all three choice situations.

### Choices Under Risk, Ambiguity, and Uncertainty

Participants made 108 description-based choices. In 54 of the gambles, they made a choice under risk, choosing between a safe gain or loss (e.g., 100% chance of winning €5) and a risky option offering some chance of a gain or loss (e.g., 50% chance of winning €10) or otherwise nothing (50% of winning €0). As in earlier developmental studies^35^,^36^, we used wheels of fortune to visually display the probabilities associated with each option (Fig. 1A). These stimuli are regularly used in developmental studies to communicate probabilities to participants not yet familiar with fractions or percentages. Another 54 of the description-based gambles represented choices under ambiguity. Here, the risky option was partly occluded (see the gray areas in Fig. 1B,C^25^). We used three levels of occlusion to quantify individuals’ sensitivity to ambiguity^16^. An ambiguity-neutral person will treat the occluded option as offering a 50% change of winning (or losing). An ambiguity-seeking (averse) person will be more optimistic (pessimistic) and act if the chance were larger (smaller) than 50%. Note that the scope of the occluded area did not change the objective probability of the ambiguous option (which was always 50% of winning).

In the choices under uncertainty task, we used the sampling paradigm to investigate developmental changes (see Fig. 1D). Respondents were confronted with two payoff distributions (gambles) with unknown outcomes and probabilities (similar to a wheel of fortune with a full occluder). Before choosing, participants could sample each distribution. Sampling revealed the possible monetary outcomes associated with each distribution; the outcomes’ occurrences signaled their relative frequencies. Samples were not consequential (i.e., there were no costs). Participants could sample as often as they desired before making a final decision (which was incentivized and thus consequential). Ceteris paribus, the more samples people drew, the more precise their mental model of the payoff distributions’ possible outcomes and relative frequencies (according to the law of large numbers^37^). All participants were presented with 10 different pairs of payoff distributions in random order. The payoff distributions were based on the classic choice situations used in previous studies^18^. These gambles typically have one option with a low-probability event (see Table S1 for full descriptions of all 10 pairs). Implementing the sampling paradigm made it possible to study two main components of an individual’s decision-making process: search effort and ultimate choice. In our analyses, we focus on search effort, as a previously under-used indicator of uncertainty attitude.

### Behavioral Analyses

#### Description-Based Choices Under Risk and Ambiguity

To examine developmental changes in risk and ambiguity aversion, we first fitted a power utility function that integrates sensitivity to different levels of ambiguity to the choice data^21^,^38^:


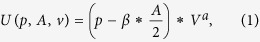


where *v* represents the amount of money that could be won, *p* the probability, *A* the ambiguity level (size of the occluder), α the risk attitude, and β the ambiguity attitude. A risk-neutral person’s α is 1; she will consistently choose the option with the highest expected value. In the gain domain, α > 1 indicates risk seeking, that is, a person who prefers the risky option to a certain one with an equal or higher expected value; α < 1 indicates risk aversion, that is, a person who prefers a certain option to a risky one with an equal or higher expected value. An ambiguity-neutral person, with β = 0, will treat all ambiguous options as equal to a 50/50 option. In the gain domain, an ambiguity-averse person (β > 0) will treat the extent of occlusion as an indicator of the decrease in the probability of winning, whereas an ambiguity-seeking person (β < 0) will act as if the probability of winning exceeds 50%. In the loss domain, the sign on the β parameter is reversed in relation to ambiguity attitude (e.g., β < 0 indicates ambiguity aversion).

**Figure 2:**
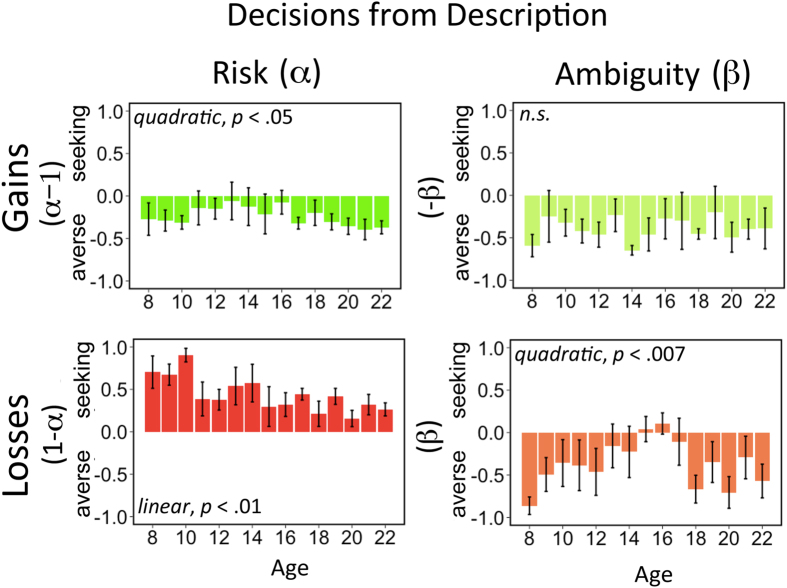
Behavioral and modeling results for risk and ambiguity attitude. Parameter estimates of risk (α) and ambiguity (β) attitudes. The parameter values were transformed such that, in each graph, values larger than zero indicate risk/ambiguity seeking and values smaller than zero indicate risk/ambiguity aversion.

To model trial-by-trial choices, we used the logistic choice rule^39^ to compute the probability (*P*_*Risky*_) of choosing the risky/ambiguous option as a function of the difference in subjective value *U*_*Risky*_ and *U*_*Safe*_(as determined by the utility function described in Eq. 2):


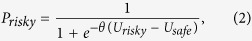


where 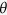 is an estimate of response stochasticity. This utility model was fitted using maximum likelihood estimation. To test its robustness, we compared it with alternative models and a model-free analysis (see Supporting Information, Table S2 and Figure S1 for details). The model fits indicated that there were no age-related differences in the degree of stochasticity in choice behavior (all *p*’s > 0.3). More importantly, participants’ choices followed the canonical pattern of risk aversion in the gain domain and risk seeking in the loss domain (Fig. 2). Replicatinga previous result^38^, we observed that risk and ambiguity attitudes were not related to each other (all *p*’s > 0.27). However, we found that choices under risk and ambiguity were correlated across valence domains (see Table S3. As our results replicated key results of previous studies, we can now turn to the developmental trends.

Figure 2 plots the developmental trends for risk and ambiguity attitudes. For risk attitudes, we found primarily linear trends, but also a quadratic trend. Specifically, in the gain domain, we obtained a linear decrease in risk attitude (*β*_*linear*_ = −0.12 95% CI = [−0.22, −0.2], *p* < 0.05) and an equally well-fitting quadratic trend (*β*_*quadratic*_ = −0.08, 95% CI = [−0.12, −0.04], *p* < 0.05; top left panel of Fig. 2 and Table S4). In the loss domain, we observed a linear decrease in risk attitudes across adolescence (*β*_*linear*_ = −0.15, 95% CI = [−0.21, −0.10], *p* < 0.01; bottom left panel of Fig. 2).

Concerning attitudes to ambiguity, Fig. 2 (right panels) shows that participants of all ages were generally ambiguity averse in both the gain and the loss domain. This finding is in line with previous reports^16^,^21^,^38^,^40^. More specifically, in the loss domain, there was a significant quadratic trend (*β*_*quadratic*_ = −0.2, 95% CI = [−0.26, −0.14], *p* < 0.007), indicating reduced ambiguity aversion in mid-adolescence (peaking at 15‒16 years). The analyses did not indicate any age-related changes in the gain domain (all *p*’s > 0.7).

#### Choices Under Uncertainty: Sampling and Choice

**Figure 3:**
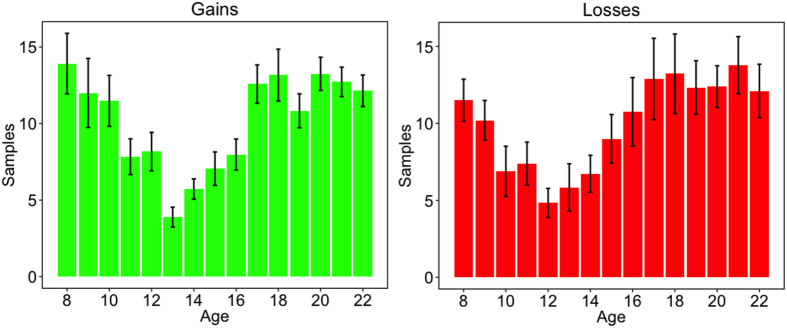
Sample sizes in choices under uncertainty. Mean number of samples per age group and separately for the gain and loss domain (error bars show standard errors).

First, we examined the mean number of samples that participants took before making a final choice. Consistent with previous results, we found that participants searched relatively little (*M* = 11.36) before choosing (see ref. 18). As Fig. 3 shows, adolescents sampled even less than children or adults did in both the gain domain (*β*_*quadratic*_ = 2.261, 95% CI = [0.790, 3.732], *p* < 0.001) and the loss domain (*β*_*quadratic*_ = 1.310, 95% CI = [0.794, 2.132], *p* < 0.02). As there was no interaction between search and feedback valence, we used the average search effort collapsed over valence domains in the subsequent analyses.

Real-world risks often have a specific structure, namely, a kind of rarity‒severity pattern. Highly detrimental events are, typically, relatively unlikely to occur. For instance, the chance of contracting an HIV infection from one unprotected sexual encounter is well under 1%^41^. The chance of experiencing such a low-probability (rare) event is a function of the amount of experience (or here, search volume). Indeed, in line with previous studies^18^, we found that our participants frequently did not experience the rare event before making a choice. Averaged across all participants and all trials, rare events were observed in just 38% of cases. As expected, the frequency of encountering the rare event was positively correlated with sample size, (*r*_pearson_ = 0.806, *p *< 0.001).

**Figure 4:**
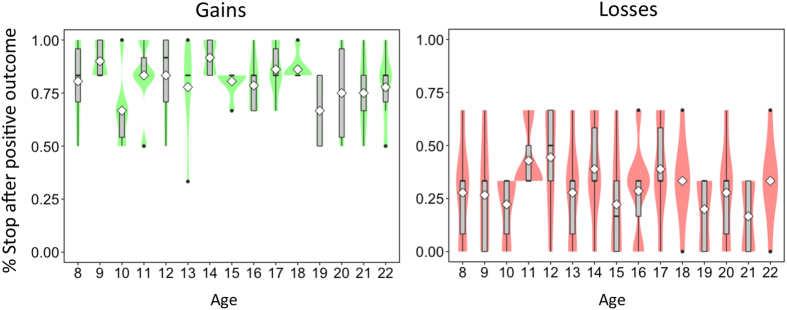
Stopping rules. Percentage of times participants stopped after encountering a positive outcome (i.e., a nonzero outcome in the gain domain or a zero outcome in the loss domain), binned by age group. White diamonds represent group means.

Next, we investigated whether there were age differences in participants’ decision to stop sampling relative to the valence of the events encountered. These analyses focused on participants’ last draw, just before they made a choice. Our analyses of respondents’ implicit stopping rules revealed a large effect of valence (β_condition_ = 0.49, p < 0.001) but no main effects (*β*_age_ = 0.004, p = 0.41) or interactions (β_age*condition_ < 0.001, p = 0.99) with age (see Fig. 4). Specifically, in the gain domain, participants more often stopped their search after encountering a positive (and nonzero) outcome. The opposite was true for the loss domain. Here, the only positive event was the zero outcome, after which people were likely to continue to sample. In other words, participants more often stopped after a nonzero outcome, regardless of whether it took the form of a gain or a loss.

Due to the developmental changes in search volume (Fig. 3), participants of different ages made their choices on the basis of very different amounts of information. Direct comparisons of choices between age groups are therefore problematic. However, our findings replicated the well-known description‒experience gap^18^ on the population level, and exploratory analyses of developmental trends suggest that increased risk taking in the experience versus description condition also peaks in mid-adolescence (see Supporting Information, Figure S2 and Table S1).

In sum, our results suggest that adolescents’ ambiguity attitude in the loss domain as well as their uncertainty attitude (as measured by search effort) in the gain and loss domain set them apart from children and adults. Adolescents are less ambiguity averse (when facing losses) and invest less in search. Although visual inspection suggests that the inflection point for decisions under ambiguity occurs later than for decision under uncertainty, the similarity in these developmental trends may indicate a common underlying cause. However, the correlation between search effort and ambiguity attitude was not significant (*r*_pearson_ = −0.17, *p = *0.45), suggesting that different psychological processes may underlie the two. To further explore this issue, we performed exploratory analyses of the influence of cognitive and motivational processes on choices under risk, ambiguity, and uncertainty.

### Cognitive Ability and Motivation

Current theoretical models emphasize that adolescent risky behavior is the result of developmental change in both cognitive and motivational processes^42^,^43^,^44^,^45^. However, their relative contribution to developmental differences in ambiguity- and uncertainty-related behavior remains unclear.

#### Cognitive Abilities

We employed three indicators of cognitive development: measures of general cognitive ability (CFT-20^46^), working memory (Digit Span^47^) and number sense (Panamath^48^). Scores on the Panamath test, which provides an estimate of approximate number sense, are strongly correlated with scores on standardized math achievement tests^49^. We therefore expected number sense to be specifically related to behavior in description-based choices (Fig. 1A), where both numerical probability and/or outcome information were explicitly stated^50^,^51^. On the other hand, previous studies have shown that working memory capacity is related to search volume (e.g. ref. 52). We therefore expected part of the age-related change in search in decisions under uncertainty (Fig. 1D) to be explained by the age-related increase in working memory capacity.

As expected, performance on all three cognitive measures increased linearly with age (Figure S3). However, none of the cognitive measures was associated with risk or ambiguity attitudes (Table S5). Although working memory showed only a trending relationship with sampling volume (*β* = 0.159, 95% CI = [0.39, 0.277], *p* = 0.09), it was positively associated with the frequency with which the rare event was seen (*β* = 0.267, 95% CI = [0.153, 0.381], *p* < 0.05; Table S6).

#### Motivation: Novelty and Intensity Seeking

We used the Arnett Inventory of Sensation Seeking (AISS) to gauge motivational development^53^. This scale consists of subscales representing novelty seeking (“*I would like to travel to places that are strange and far away*”) and intensity seeking (“*I would like to gamble with money, if I could afford it*”). Both constructs are independently associated with real-world risk taking and scores tend to peak in adolescence^54^,^55^. Therefore, they can be expected to be associated with preference for ambiguity, and also with adolescents’ decreased search effort.

As expected, novelty seeking peaked in mid-adolescence. Contrary to previous findings, however, we did not find developmental differences in intensity seeking (Figure S3). More importantly, novelty seeking was positively correlated with ambiguity seeking in the loss domain (*β* = 0.141, 95% CI = [0.78, 0.204], *p* < 0.05; Table S8). Interestingly, novelty seeking also proved to be negatively correlated with search effort (*β* = 0.296, 95% CI = [0.162, 0.376], *p* < 0.05; Table S9).

To conclude, given the exploratory nature of the analyses, these observations should be interpreted with caution. Nevertheless, they suggest that developmental change in ambiguity and uncertainty attitudes may be driven primarily by developmental change in motivational processes and less so by change in cognitive processes. However, behavior in two choice situations—choice under ambiguity and choice under uncertainty—was not correlated across individuals. This finding suggests that there may be specific motivational processes, and/or interactions with other cognitive variables, that mediate the relationship between novelty seeking and different forms of attitudes toward the unknown (see Table S7 for correlations between motivational and cognitive variables).

### Real-World Risk Taking

Our within-subject design afforded us the unique opportunity to compare how the behaviors measured in three different choice situations—representing risk, ambiguity, and uncertainty— are differentially associated with that of self-reported real-world risk behavior. Many previous studies have used only a single type of choice situation or failed to collect self-report measures of real-world risk behavior. We used the Adolescent Risk Questionnaire (ARQ^25^) to collect data on self-reported risk behavior from a subset of our sample (ages 11‒18). The ARQ is a well-validated questionnaire for adolescents aged between 11 and 18 (for German adolescents, see ref. 56) and has been associated with measures of risk^57^ and ambiguity^20^. We focus here on self-reported risk behavior, but corresponding analyses for risk attitudes are reported in the Supporting Information (Tables S10 and S11).

Bringing self-reported risk behavior and the experimental behaviors together, we found that adolescents who reported engaging more frequently in reckless (*β* = 0.158, 95% CI = [0.76, 0.234], *p* < 0.03) and rebellious (*β* = 0.241, 95% CI = [0.167, 0.321], *p* < 0.008) behaviors were also significantly more tolerant of ambiguity in the loss domain (Table 1). For risk attitude, in contrast, no such relationship emerged, in either the gain or the loss domain (Table 1). That results were significant only in the loss domain is perhaps not surprising, as almost all items in the ARQ relate to activities or events with potentially negative (and not positive) outcomes. Equally important, we found that teenagers who searched little before making a sample-informed choice showed higher values on the thrill-seeking subscale of the ARQ (*β* = −0.326, 95% CI = [−0.459, −0.193], *p* < 0.01; Table 2). Finally, as expected, we found that novelty seeking, but none of the cognitive measures, was correlated with self-reported risk behavior (see Figure S5 for details); these findings thus offered a good validity check of the self-reported risk behaviors.

To conclude, both ambiguity attitude and uncertainty attitude, measured behaviorally (Fig. 1), proved to be good predictors of different, and uncorrelated, risk behaviors. Ambiguity attitude was more strongly associated with more severe and rebellious forms of risky behavior, such as using drugs and having unprotected sex, whereas uncertainty attitude was associated with less severe and more socially accepted risky behaviors, such as skiing and parachute jumping.

## Discussion

Adolescence is characterized by a spike in mortality and morbidity rates, largely due to adolescents’ willing engagement in risky behaviors^1^,^2^,^3^,^4^. However, identifying a parallel spike in adolescent risk taking in the behavioral choice situations implemented in psychologists’ and economists’ laboratories has remained a challenge^11^. This incongruity may result partly from the micro-world of the laboratory being an inadequate proxy for real-world risk behaviors. One dimension on which the two worlds diverge is in the extent of individuals’ knowledge about the potential outcomes of risky actions and their probabilities. When adolescents “push their luck”—by experimenting with drugs or having unprotected sex, for example—their knowledge of the possible consequences and their respective probabilities is likely to be vague. Furthermore, they have the opportunity to search for more information before deciding to embark on risky behaviors. Might behavioral tasks that reflect decision makers’ limited knowledge and potential agency in real-world situations be better suited to tracking adolescent risk taking? To address this question, we administered well-established experimental paradigms to gauge, in parallel, developing attitudes to risk, ambiguity, and uncertainty across adolescence.

Our results indicate that these attitudes have different developmental trajectories and, importantly, may have distinct predictive potential. As expected and as previously observed, we found that participants’ attitudes involving risk (known outcomes and probabilities) changed linearly from childhood to adulthood. However, ambiguity attitudes (known outcomes but unknown probabilities) showed nonlinear developmental trends, with ambiguity tolerance peaking in adolescence. This finding is in line with two previous studies that also found adolescents to be more tolerant of ambiguity than adults^20^,^22^, but contradicts another recent study suggesting that children may be even more ambiguity tolerant^24^. More importantly, we found a similar nonlinear developmental trajectory in respondents’ uncertainty attitude. Both ambiguity and uncertainty attitudes—but not risk attitude—were correlated with self-reported risk behavior. These results confirm the observation that the risk attitude—as elicited by description-based options that fully inform the decision maker about outcomes and probabilities—is not a good reflection of real-world risk and people’s decision to engage with it. Ambiguity and uncertainty attitudes, in contrast, more closely approximated the nonlinear developmental pattern of real-world risk taking (inverted U). However, ambiguity and uncertainty attitudes were not correlated across participants, and we also found ambiguity and uncertainty attitudes to be associated with distinct types of self-reported risky behavior. Ambiguity was related to potentially more serious risk taking (e.g., drug taking), whereas uncertainty was correlated with more benign and socially accepted forms of risk taking (e.g., skiing). Thus, although both attitudes have comparable developmental trajectories, they explain unique variance in risky behaviors. These results suggest that what is unique about adolescent risk taking is a willingness to engage in actions with unknown consequences, as well as lower motivation to search for information that reduces uncertainty. These results suggest new approaches to policy interventions (see below) but, at the same time, they raise the question about the origin of these unique attitudes.

Developmental frameworks of risky behavior—i.e., fuzzy trace theory^4^ and imbalance or dual-process theories^43^,^44^,^58^,^59^—attribute adolescent risk taking to the development of multiple interacting cognitive and motivational processes. However, they make different predictions. The imbalance view predicts that risk-taking propensities peak in early/mid-adolescence, whereas fuzzy trace theory is more consistent with a gradual decline in risk taking (see Defoe *et al*.^11^). Interestingly, our data suggest that both predictions may be valid. Whereas fuzzy trace theory predicts the linear developmental trajectory we observed for risk attitude (i.e., a gradual increase in risk aversion; Fig. 2), imbalance models are better able to capture the developmental trajectory of uncertainty and ambiguity attitude. In contrast to the predictions of these frameworks, however, our results suggest that risky behavior is associated with developmental changes in motivational (specifically, novelty-seeking) but not cognitive processes. More specifically, our results are consistent with the long-held belief that adolescents’ risky behavior is fueled by their distinct motivation to seek new experiences^60^.

Our findings suggest new directions for policy interventions. At first glance, they could be taken to suggest that providing information on relevant outcomes and their probabilities, in ways that render information search unnecessary, would be a successful prevention strategy. However, the evidence for success of information campaigns such as the Drug Abuse Resistance Education (DARE) program is rather discouraging^61^. Indeed, it may be precisely *because* adolescents are willing to leap before they look that these campaigns are relatively ineffective. Instead of, or in addition to, providing information, it may be more successful to afford adolescents (virtual) experience, including the experience of rare, consequential events^62^,^63^.

The present findings suggest interesting future directions for research. First, eliciting choices under risk may have limited value in revealing the dynamics behind risky adolescent behavior. Instead, choice situations should be administered in the context of behavioral paradigms that capture key aspects of real-world decision making, such as ignorance, vague information, and agency (representative design^64^). Specifically, the role of agency and learning has been underrepresented in developmental research. Second, several recent studies have shown that adolescents are more likely to engage in risky behavior when in the company of friends and peers than when alone^65^. A better understanding of how social context and peer pressure shapes individual approaches to ambiguity or uncertainty is therefore also important (e.g., competition has been shown to drastically curtail exploratory search^66^). Third, developmental differences in learning from feedback have been documented^29^,^67^,^68^. To predict how risky behavior emerges in contexts of uncertainty, researchers therefore may also need to trace developmental changes in belief updating. Fourth, our results suggest that no single measure of behavioral risk taking will suffice to capture the various manifestations—some more dangerous and undesirable than others—of adolescent risk taking. In order to predict different aspects of risk taking in the real world, it seems promising to combine various behavioral measures rather than betting on a single one. To conclude, our results suggest that research should move toward more representative design (s) and a multidimensional measurement of risk.

## Materials and Methods

### Participants

We recruited 105 participants (56 female) between 8 and 22 years of age (*M* = 14.87, *SD* = 4.27). Participants received €10 per hour for their participation, plus potential additional earnings resulting from their choices in the decision tasks (see below). The Ethics Committee of the Max Planck Institute for Human Development approved the study, and the experiment was performed in accordance with all relevant guidelines and regulations. All adult participants gave written, informed consent before completing the task. All under-aged participants (<18 years) were accompanied by a parent/caregiver who also signed the consent form.

### Behavioral Measures and Self-Report

Participants received extensive instructions and training and had to pass a detailed comprehension check before the tasks were administered. They were seated in front of a computer screen, on which all tasks and questionnaires were displayed. Participants were instructed that they could take breaks between tasks for as long as they wanted and that they could ask the experimenter questions at any time.

In the risk and ambiguity tasks, participants were confronted with 108 monetary gambles. In 54 of these gambles, they chose between a certain amount of money (e.g., 100% chance of winning €4) or a lottery offering some chance of winning (e.g., 50% of winning €8; otherwise nothing); in the other 54 gambles, the lottery option was partly occluded^20^.

The properties of the lottery were varied systematically (in random order) across trials to determine how choice was affected by the probability of winning (from 10% to 90% in steps of 10%), the magnitude of the potential gain (€3, €4, 8, €16, and €32), and ambiguity about the probability of winning (20%, 50%, and 80% ambiguity). The monetary amounts and probabilities were based on earlier studies (e.g ref. 18). Half the trials were loss trials, using the same monetary amounts but the opposite sign. In addition to these gambles, we included 10 decision problems frequently used in research on decisions under uncertainty. We also included 10 control trials in which the amount of the safe option was equal to that of the risky option (these were all in the gain domain to ensure that participants’ total payoff was always positive). All gambles were presented in random order (full list available upon request).

Importantly, there was no age-related difference in choice pattern in the control condition, where 94% of choices did not violate first-order stochastic dominance (i.e., participants did not select the option with same outcome but lower probability) (*β*_*linear*_ = 0.08, 95% CI = [−0.06, 0.24], *p* < 0.274; *β*_*quadratic*_ = 0.05, 95% CI = [−0.02, 0.12], *p* < 0.174; *β*_*emerging*_ = −0.03, 95% CI = [−0.09, 0.04], *p* < 0.316). Consequently, no participants had to be excluded based on their choice pattern. All participants were informed that all choices were incentivized. They were not told what the exact exchange rate would be, but were informed that they could earn an additional amount between €0 and €6. There were no measurable order effects on task behaviour or parameter estimates. Finally, both tasks and instructions were extensively pilot-tested on children (8‒11 years) to make sure that the youngest participants understood the task.

After the experimental sessions, participants took a break before providing demographic information, completing the AISS, and performing several psychological tests (CFT-20, Panamath, and Digit Span). Finally, participants aged between 11 and 18 years completed the ARQ. All participants were paid €10 per hour (in the case of under-aged participants, the parents received the money), plus the performance-related bonus (on average, €3.40).

## Additional Information

**How to cite this article**: van den Bos, W. and Hertwig, R. Adolescents display distinctive tolerance to ambiguity and to uncertainty during risky decision making. *Sci. Rep.* **7**, 40962; doi: 10.1038/srep40962 (2017).

**Publisher's note:** Springer Nature remains neutral with regard to jurisdictional claims in published maps and institutional affiliations.

## Supplementary Material

Supplementary Information

## Figures and Tables

**Table 1:** ARQ risky behavior: results of logistic regression with risk and ambiguity attitudes across gain and loss domains as dependent variables.

	Risk		Ambiguity	
	Gain	Loss	Gain	Loss
Age	0.105 (0.254)		0.061 (0.145)	
Age^2^				−0.751** (0.273)
Age^3^		−0.219 (0.180)*		
Gender	−0.062 (0.133)	0.058 (0.169)	0.080 (0.145)	0.091 (0.145)
Thrill seeking	0.047 (0.069)	0.043 (0.090)	0.007 (0.075)	−0.069 (0.075)
Rebellious	0.088 (0.072)	0.103 (0.088)	0.050 (0.079)	0.158* (0.076)
Reckless	−0.023 (0.071)	0.052 (0.090)	0.030 (0.080)	0.241** (0.077)
Antisocial	−0.126 (0.068)	−0.085 (0.087)	0.062 (0.074)	0.036 (0.074)
Constant	−0.062 (0.117)	0.251* (0.119)	−0.348** (0.102)	−0.038 (0.126)

Regression models included age and gender (1 = male, 0 = female). The best-fitting age trend for each dependent variable is reported. Each model contains the ARQ subscales of risk behavior. Note. ^*^p < 0.05; ^**^p < 0.01 (standard errors in parentheses).

**Table 2:** ARQ risky behavior: results of logistic regression with sampling behavior in decisions under uncertainty as the dependent variable.

	No. samples	% rare event seen
Age		0.223 (0.288)
Age^2^	0.245 (0.486)	
Gender	−0.465 (0.259)	−0.490 (0.297)
Thrill seeking	−0.326* (0.133)	0.280 (0.157)
Rebellious	−0.124 (0.135)	0.167 (0.156)
Reckless	−0.012 (0.138)	−0.108 (0.160)
Antisocial	−0.056 (0.134)	−0.089 (0.152)
Constant	−0.084 (0.230)	0.245 (0.208)

Regression models included age and gender (1 = male, 0 = female). The best-fitting age trend for each dependent variable is reported. Each model contains the ARQ subscales of risk behavior. Note. *p < 0.05; **p < 0.01 (standard errors in parentheses).
