# Supplementary material for: Adolescents display distinctive tolerance to ambiguity and to uncertainty during risky decision making
Source: Sci Rep. 2017 Jan 18;7:40962. doi: 10.1038/srep40962 (PMC5241878; doi:10.1038/srep40962)
Supplement: Supplementary Information [file srep40962-s1.doc]

**SUUPLEMENTARY INFORMATION**

Adolescents display distinctive tolerance to ambiguity and to uncertainty during risky decision making

Wouter van den Bos^a,1^ & Ralph Hertwig^a^

^a^Center for Adaptive Rationality, Max Planck Institute for Human Development,
D-14195 Berlin, Germany

.

**SUPPORTING INFORMATION**

**Methods**

*Model Fitting Procedure*

We used the simplex algorithm of the general-purpose optimization toolbox (optim) in R for model ﬁtting. This function assumes that each individual will choose the option with the highest subjective value with highest probability. Individual parameter estimates for each of the models were determined as those that maximized the likelihood of the observed data. More specifically, for each participant, the fitting procedure was preceded by a grid search in order to circumvent possible local minima. Additionally, for the minimization function, the Log (Likelihood) was transformed to G^2^  (= ‒2*Log(Like)). The model was fit separately for the gain and loss domains. We did not find any developmental differences in response noise in either domain (all trends *p*s > .62 ).

To benchmark the performance of the utility model, we fitted two alternative models and a random choice model (*p_Risky_* = .5). The first model suggests that the subjective probability of the ambiguous option is exponentially modulated by the size of the occluder; it proved to be a superior fit in the study by Hsu and colleagues ^25,84^ :

Eq. S2
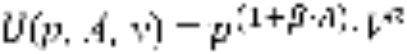


The second model simply assumes that all levels of ambiguity are treated the same:

Eq. S3
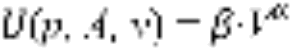


The two models were fitted using the same choice function as described in Eq. S1. For each model, the individually estimated G^2^ was smaller than the G^2^ of the random choice model. Further comparisons revealed that the linear model (Eq. 1) fit the behavioral data the best (see below). It was therefore used in all subsequent analyses. Finally, we compared the linear model with a model-free analysis of the choice data, which revealed behavioral patterns that were entirely consistent with the modeling results (see Figure S3).

*Model-Free Analyses*

Following Defoe and colleagues ^11^, we defined risk taking behaviorally as choosing the option with the highest outcome variability. In the context of our design, that was identical with *not* choosing the safe (100%) option. To map the risk attitudes of participants based on their behavior alone, we compared their choices with a risk-neutral baseline. A risk-neutral participant would choose the risky option only if it were the option with the highest expected value (EV; probability × outcome). In our experiment, a risk-neutral strategy would lead to 56% risky choices in the gain domain and 35% risky choices in the loss domain. Participants were considered risk seeking if they chose the risky option more often than defined by the risk-neutral benchmark and as risk averse if they chose it less often. As expected, our results followed the canonical pattern of risk aversion in the gain domain and risk seeking in the loss domain (83; see Figure S1). Furthermore, we found a developmental decrease in risk seeking (loss domain: *β_linear_* = ‒5.2, 95% CI = [‒8.87, ‒1.52], *p* < .005; *β_emerging_* = ‒2.8, 95% CI = [‒4.6, ‒1.1], *p* < .004; Table S3), but only a trending relationship between age and risk aversion (gain domain: *β_quadratic_* = 2.09, 95% CI = [‒.22, 4.41], *p* = .12).

An ambiguity-neutral person is expected to treat ambiguous gambles the same as 50% risky prospects. We therefore behaviorally defined ambiguity seeking as choosing the ambiguous prospect more often than 50/50 gambles, and ambiguity averse as choosing the ambiguous prospect less often than 50/50 gambles. Thus, for each participant, we directly compared the proportion of risky choices in all 50/50 gambles with the proportion of ambiguous choices. In line with earlier studies and our modeling results, we found that all participants were generally ambiguity averse in both domains (Figure S1). For losses, there was also a significant quadratic trend, *β_quadratic_* = 11.02, 95% CI = [6.96, 15.08], *p* < .007, indicating reduced ambiguity aversion in mid-adolescence. For gains, in contrast, our results did not indicate age-related changes (all *p*’s >.6).

**Figure S1.** Model-free analyses of choice behavior. For the calculation of the risk and ambiguity attitudes as presented on the y-axis please see text.


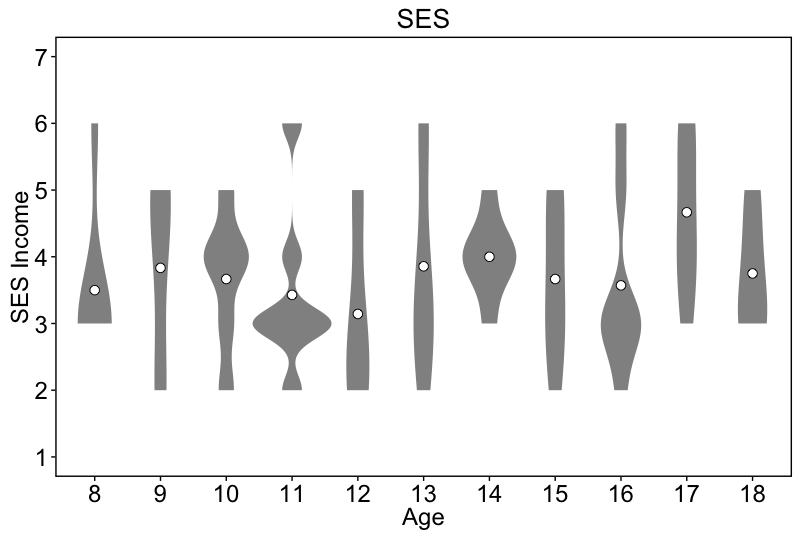


Figure SX. SES distrinution per age group, open circle represents mean.

*Description Experience Gap*

Studies employing the sampling paradigm have consistently shown that people have different risk preferences for experienced gambles than they do for matched gambles that are fully described (the description‒experience gap; ). More specifically, people making decisions from experience tend to be more risk taking when the rare event is an undesirable outcome, and less risk taking when it is desirable. Following earlier studies, we compared the percentage of risky options chosen in experience-based gambles and matched description-based gambles (aggregated across all age ranges). The description-based gambles were a subset of the gambles in the risky-choice paradigm reported above. We found a significant gap between description and experience in 7 out of 10 problems (see Table 1), all in the expected direction.

**Figure S2.** Developmental differences in decisions from experience

Change in proportion of risky choices in the sampling condition relative to the same gambles in the description condition, broken down by the desirability of the rare event.

To further explore age‒related differences in choice behavior, we pooled all decisions according to the desirability of the rare event and investigated the description‒experience gap as a function of age. For choices with undesirable rare events, these analyses revealed a quadratic trend in the size of the gap (*β_quadratic_* = ‒.165, 95% CI = [‒.246, ‒.083], *p* < .001; Figure S3), with a peak in mid‒adolescence. When the rare event was desirable, in contrast, there was only a trending quadratic relationship with age (*β_quadratic_* = ‒.065, 95% CI = [‒.136, .006], *p* = .07; Figure S2). Thus, particularly adolescents seem underweight the probability of the rare event in situations where they can experience probabilities than in situations where probabilities are presented. Of course, one difficulty in interpreting these developmental trends is that these decisions were based on different experiences. To make them more comparable, we ran the same analyses on a subset of problems in which participants saw the rare events at least once (Figure S3C and S3D). Consistent with our other findings, these analyses revealed a quadratic trend in the size of the gap for choices with undesirable rare events (*β_quadratic_* = ‒.250, 95% CI = [‒.361, ‒.140], *p* < .001; Figure S5), with a peak in mid‒adolescence. But, again, when the rare event was desirable, there was only a trending quadratic relationship with age (*β_quadratic_* = ‒.078, 95% CI = [‒.166, .009], *p* = .09).

Finally, we considered to what extent participants chose the option with the highest mean reward, conditional on what they experienced in the task. To this end, we calculated, separately for each individual, the mean reward for each option by dividing the total sum of experienced outcomes by the number of sampled outcomes (the search volume). There was a trending linear increasing trend (*β_linear_* = .031, 95% CI = [.008, ‒.070], *p* = .12), suggesting that, as participants get older, the more their behavior is consistent with maximization of expected reward (based on their experience).

**Figure S3. Developmental differences in cognitive ability and sensation seeking**

**Upper panels)** CFT-20, average Digit Span, and Panamath scores increased linearly with age (*β_CFT_* = .263, 95% CI = [.062, .464], *p* < .011; *β_Digit_* _Span_ = .259, 95% CI = [.054, .464], *p* < .015; *β_Panamath_* = ‒.312, 95% CI = [‒.510, ‒.115], *p* < .002)**. Lower panels)** Novelty seeking peaked in mid-adolescence (*β_Novel_* = ‒.277, 95% CI = (‒.501, ‒.054], *p* < .017), but the intensity scale did not change with age (*β_Intens_* = ‒.130, 95% CI = (‒.336, .077], *p* < .22). All significant trends are reported.

**
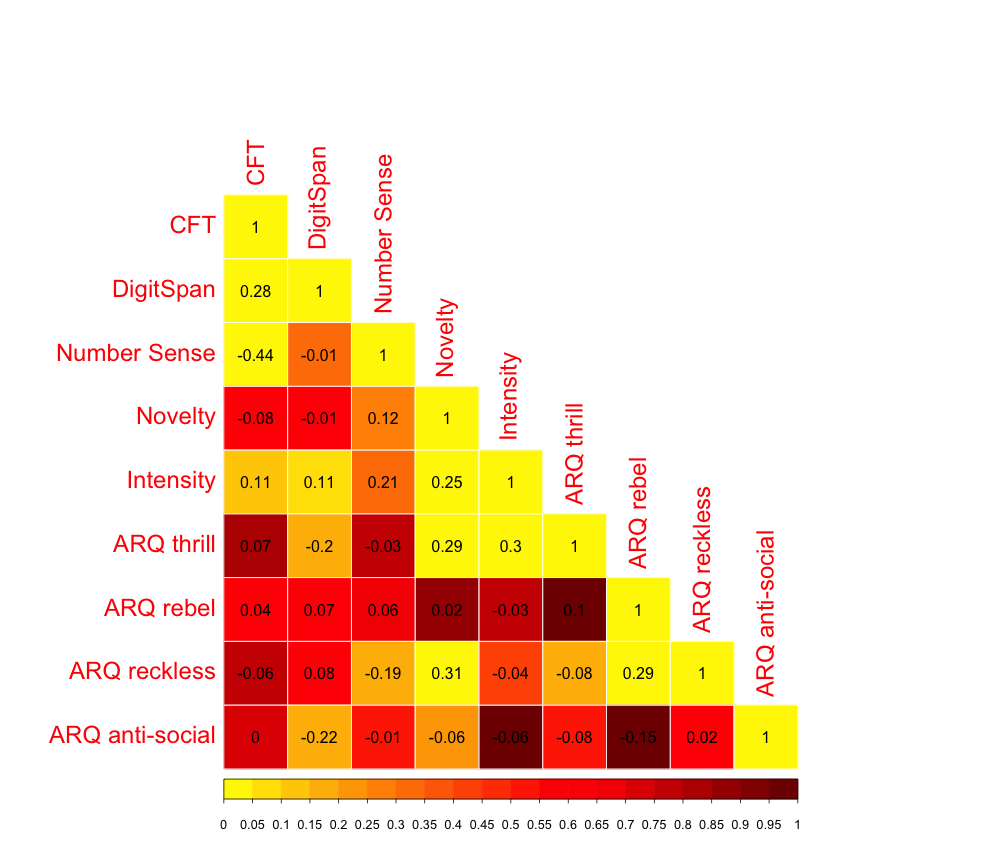
**

**Figure S4. Sensitivity analyses**

Correlations between cognitive measures, self-reported sensation seeking, and risk behavior. Tile numbers represent Pearson’s *r* and the color represents the *p* value. As expected, we found that (i) cognitive ability (CFT) is correlated with both digit span and number sense, and (ii**)** sensation seeking is a good predictor of some types of risk behavior.

**Table S1**. Gambles used in the choices under uncertainty task

|  | Option 1 | | | Option 2 | | | % risky options chosen | |  | |
| --- | --- | --- | --- | --- | --- | --- | --- | --- | --- | --- |
| Rare event desirable | Prob. | Payoff | Prob. | | Payoff | Experience | | Description | | χ^2^ |
| Yes | 10% | 32 | 100% | | 3 | 20% | | 56% | | 22.30*** |
| No | 10% | ‒32 | 100% | | ‒3 | 75% | | 59% | | 4.91* |
| Yes | 100% | ‒3 | 80% | | ‒4 | 35% | | 49% | | 3.24 |
| No | 100% | 3 | 80% | | 4 | 61% | | 61% | | 0.1 |
| Yes | 90% | ‒10 | 100% | | ‒9 | 32% | | 71% | | 25.45*** |
| No | 90% | 10 | 100% | | 9 | 59% | | 63% | | .21 |
| Yes | 10% | 10 | 100% | | 1 | 32% | | 50% | | 5.08* |
| Yes | 10% | 32 | 100% | | 10 | 8% | | 28% | | 10.45*** |
| No | 70% | 4 | 30% | | 6 | 29% | | 12% | | 7.48** |
| N.A. | 60% | 4 | 40% | | 6 | 63% | | 42% | | 7.15** |

*Note.* Set of gambles used for decisions under risk and decisions under uncertainty the decision from experience and decision from description paradigms. The decision‒experience gap is described in terms of difference in the propensity to take the risky option between conditions, which was tested using χ^2^: *p < .05;*******p* < .01** and *** *p* < .001.

**Table S2: Model fits for different ambiguity models (G^2^)**

|  | Gains | Losses |
| --- | --- | --- |
| Simple | 7247 | 7324 |
| Linear | 7203 | 7312 |
| Non-linear | 7205 | 7314 |

*Note.* For each model, the G^2^ is reported given that all models have an equal number of parameters.

**Table S3. Correlation between risk attitude and ambiguity attitude**

*Note.* Correlation plot parameter estimates of Decisions under Risk and Decisions under Ambiguity. R squared values are reported; * *p* < .05.

**Table S4. Developmental differences in model-based risk (α) and ambiguity (β) attitudes**

*Note.* In case of multiple significant age trends we report model fits for model comparisons. Model fits for each age trend are expressed in terms of Bayesian Information Criterion (BIC), and are meaningful only within, not between, columns. BICs reported only when at least one significant trend was observed. Best fitting age trends indicated in bold.

**Table S5. Cognitive abilities: results of logistic regression with risk and ambiguity attitudes as dependent variables**

*Note.* ***p* < .01; ****p* < .001 (standard errors in parentheses).

**Table S6. Cognitive abilities: results of logistic regression with decisions from experience measures as dependent variables**

*Note.* **p* < .05. (standard errors in parentheses).

**Table S7. Correlations between cognitive capabilities and motivation scores**

*Note.* **p* < .05; ***p* < .01.

**Table S8. Motivation: results of logistic regression with risk and ambiguity attitudes as dependent variables**

*Note.* **p* < .05. (standard errors in parentheses)

**Table S9. Motivation: results of logistic regression with decision from experience measures as dependent variables**

*Note.* **p* < .05. (standard errors in parentheses)

**Table S10. ARQ Risk Perception: results of logistic regression with risk and ambiguity attitudes as dependent variable**

*Note.* **p* < .05; ***p* < .01. (standard errors in parentheses)

**Table S11. ARQ Risk Perception: results of logistic regression with decision from experience measures as dependent variable**

*Note.* **p* < .05; ***p* < .01. (standard errors in parentheses)
